# The Sequence Characteristics and Binding Properties of the Odorant-Binding Protein 2 of *Euplatypus parallelus* to Semiochemicals

**DOI:** 10.3390/ijms24021714

**Published:** 2023-01-15

**Authors:** Guangchao Cui, Xiang Zhou, Qian Wang, Kai Zhang, Lei Qin, Jixing Guo

**Affiliations:** Key Laboratory of Green Prevention and Control of Tropical Plant Diseases and Pests (Ministry of Education), School of Plant Protection, Hainan University, Haikou 570228, China

**Keywords:** odorant-binding proteins, *Euplatypus parallelus*, binding ability, fluorescence competitive binding assays, molecular docking

## Abstract

*Euplatypus parallelus* is one of the dominant rubber bark beetle species in Hainan’s rubber-planting area. Semiochemicals, including the volatiles found in rubber trees and aggregation pheromones, play an important role in the search for suitable host plants. To examine the possible functional role of highly expressed odorant-binding protein 2 of *Euplatypus parallelus* (EparOBP2) in the semiochemical recognition process, we cloned and analyzed the cDNA sequence of EparOBP2. The results showed that EparOBP2 contains an open reading frame (ORF) of 393 bp that encodes 130 amino acids, including a 21-amino-acid residue signal peptide at the N-terminus. The matured EparOBP2 protein consists of seven α-helices, creating an open binding pocket and three disulfide bridges. The results of the fluorescence binding assay showed that EparOBP2 had high binding ability with α-pinene and myrcene. The docking results confirmed that the interactions of α-pinene and myrcene with EparOBP2 were primarily achieved through hydrophobic interactions. This study provides evidence that EparOBP2 may be involved in the chemoreception of semiochemicals and that it can successfully contribute to the integrated management of *E. parallelus*.

## 1. Introduction

*Euplatypus parallelus* (Coleoptera: Curculionidae) is a destructive and widely distributed species of invasive regulated pests [1]. This species is native to Central and South America, and it seems to be spreading rapidly across Asia, Africa, and the Americas through commercial trade pathways [2,3]. The Exit–Entry and Quarantine system frequently detects and identifies this pest in imported wood, wooden crates, or pallets used to transport imported goods. *E. parallelus* has invaded both Taiwan and Hainan, China [4]. Tang et al. predicted that the northern coast of Hainan Province and the southwestern coast of Taiwan Province would be highly suitable areas for this species [5].

The *Hevea brasiliensis* rubber tree is the most important plant cultivated for rubber production in Hainan. *E. parallelus*, which has been reported to attack live rubber trees in Brazil and India, is also a dominant rubber bark beetle species found in the Hainan rubber-planting area [4,6,7,8,9]. In the search for suitable host plants, semiochemicals—including the volatiles found in rubber trees and the aggregation pheromones released by beetles that have already attacked the tree—play an important role [10,11,12,13]. A total of 27 components have been identified from the volatiles obtained from rubber trees [14]. Longifolene, tetradecane, and 2-phenyl-2-propanol have shown an attraction effect on *E. parallelus* [7,15]. Trapping experiments carried out to collect the aggregation pheromones have revealed that α-pinene, myrcene, 2-methyl-3-buten-2-ol, S-(-)-limonene, camphene, and (S)-cis-verbenol also have attraction effects on rubber bark beetles [16,17]. Because of the ecological and economic importance of rubber bark beetles, a comprehensive research base built on chemical ecology and olfactory physiology has been established. However, information on the molecular elements of odor recognition continues to be scarce [18,19].

The olfactory sense drives the behavior of bark beetles [20,21]. Insects can detect molecular information via odors in their surroundings and respond appropriately [22]. The odorant recognition process in insects is an extremely sophisticated chain reaction [23,24]. Odorant-binding proteins (OBPs), which are small, water-soluble proteins, are mainly responsible for the binding and transport of water-insoluble compounds [25]. The connectivity of disulfide bridges creates an interior hydrophobic pocket for lipophilic ligand binding. Our previous study identified 14 odorant-binding proteins based on *E. parallelus* transcriptome data. EparOBP2 was highly expressed in adults [26]. To examine the possible functional role of EparOBP2 in the semiochemical recognition process, we cloned and analyzed the cDNA sequence of EparOBP2 and the binding activities of EparOBP2 to volatiles, and both aggregation pheromones as well as the critical amino acid residues that contribute to their binding interactions were characterized. This study provides evidence that EparOBP2 might be involved in the chemoreception of semiochemicals, and that it can successfully contribute to the integrated treatment of *E. parallelus*.

## 2. Results

### 2.1. Sequence Analysis of EparOBP2

**Figure 1:**
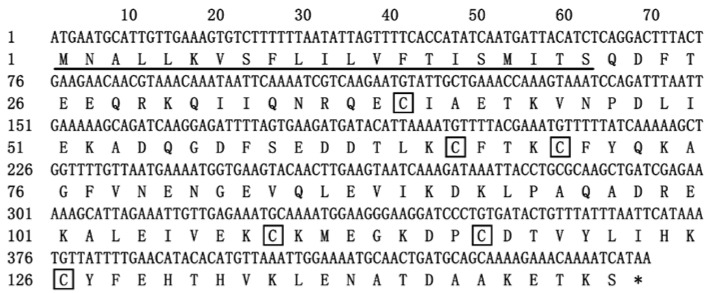
The cDNA and deduced amino acid sequences of EparOBP2. Underlines indicate the predicted signal peptides. The conserved Cys sites are indicated in black boxes. The asterisk indicates the translation–termination codon.

**Figure 2:**
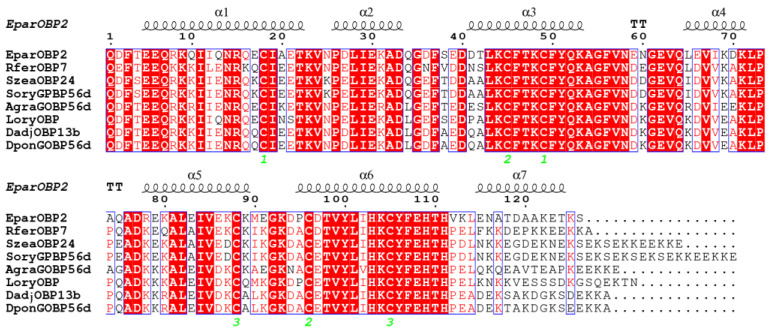
Alignment of EparOBP2 with OBP genes from other Curculionidae insects. *Anthonomus grandis* (Agra), *Dendroctonus ponderosae* (Dpon), *Dendroctonus adjunct* (Dadj), *Lissorhoptrus oryzophilus* (Lory), *Rhynchophorus ferrugineus* (Rfer), *Sitophilus oryzae* (Sory), *Sitophilus zeamais* (Szea).

The EparOBP2 cDNA sequence had a 393 bp open reading frame (ORF) and encoded 130 amino acids, including a predicted N-terminal signal peptide that was 21 amino acid residues in length (Figure 1). The molecular mass and acidic isoelectric point of the predicted mature proteins were 16.87 kDa and 5.04, respectively. EparOBP2 was found to be most similar to the general odorant-binding protein (GOBP) of *Sitophilus oryzae* (74.56%). The estimated and displayed hydropathic nature of EparOBP2 for each residue revealed that the grand average hydropathicity was −0.49. According to SOPMA analysis, the α-helix was the predominant structure of EparOBP2 (70.07%), followed by random coils (23.13%), turns (4.08%), and extended strands (2.72%) (Figure 2). This protein had a secondary structure composed of seven α-helices positioned between Glu5 and Thr22 (α1), Pro26 and Asp33 (α2), Asp41 and Lys53 (α3), Leu65 and Leu72 (α4), Arg78 and Lys89 (α5), Pro95 and Thr110 (α6), and Glu115 and Thr124 (α17) (Figure 2).

### 2.2. Sequence Alignment and Phylogenetic Analysis

We performed an amino acid sequence alignment of EparOBP2 using seven similar OBPs from other Curculionidae insects. The conserved six-cysteine signature of EparOBP2 was as follows: X_38_-Cys-X_26_-Cys-X_3_-Cys-X_38_-Cys-X_7_-Cys-X_8_-Cys-X_21_ (Figure 2). The helix framework was stabilized using three highly conserved internal disulfide bridges comprising six cysteine residues: Cys18–Cys49, Cys45–Cys96, and Cys88–Cys105.

**Figure 3:**
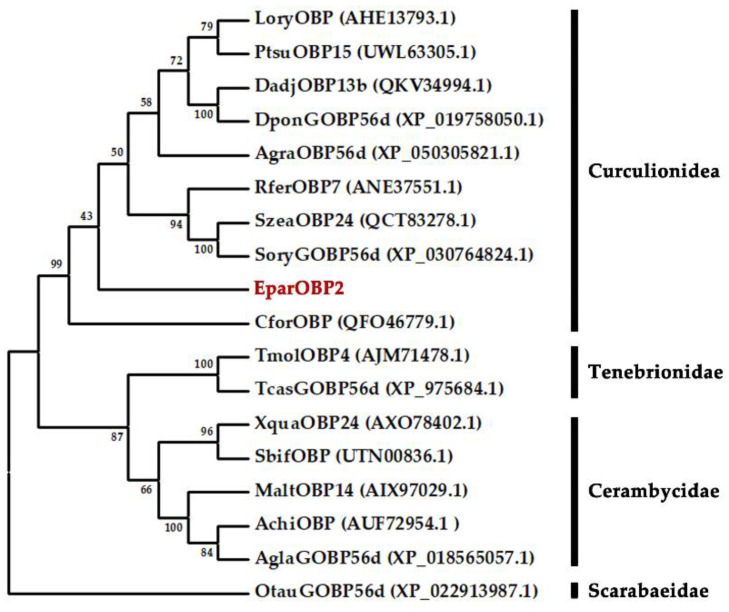
Phylogenetic tree of EparOBP2 amino acid sequences with OBP from other coleopteran insect species. The tree was constructed using the neighbor-joining method with a bootstrap of 1000 replicates. OtauOPB from *Onthophagus taurus* (Coleoptera: Scarabaeidae) was used as an outgroup to root the tree. *Anoplophora chinensis* (Achi), *Anthonomus grandis* (Agra), *Anoplophora glabripennis* (Agla), *Cylas formicarius* (Cfor), *Dendroctonus ponderosae* (Dpon), *Dendroctonus adjunct* (Dadj), *Lissorhoptrus oryzophilus* (Lory), *Monochamus alternatus* (Malt), *Onthophagus taurus* (Otau), *Pagiophloeus tsushimanus* (Ptsu), *Rhynchophorus ferrugineus* (Rfer), *Sitophilus oryzae* (Sory), *Sitophilus zeamais* (Szea), *Semanotus bifasciatus* (Sbif), *Tribolium castaneum* (Tcas), *Tenebrio molitor* (Tmol), *Xylotrechus quadripes* (Xqua).

A phylogenetic tree was constructed using the EparOBP2 and 19 OBP sequences of other Coleoptera species to evaluate the evolutionary relationships between proteins. EparOBP2 was clustered with CforOBP (QFO46779.1), SoryGOBP56d (XP_030764824.1), SzeaOBP24 (QCT83278.1), RferOBP7 (ANE37551.1), AgraOBP56d (XP_050305821.1), LoryOBP (AHE13793.1), PtsuOBP15 (UWL63305.1), DponGOBP56d (XP_019758050.1), and DadjOBP13b (QKV34994.1) in the Curculionidae superfamily, and it was determined that EparOBP2 was close to the protein SoryGOBP56d (XP_030764824.1), with 74.56% similarity (Figure 3).

### 2.3. Three-Dimensional (3D) Modeling

**Figure 4:**
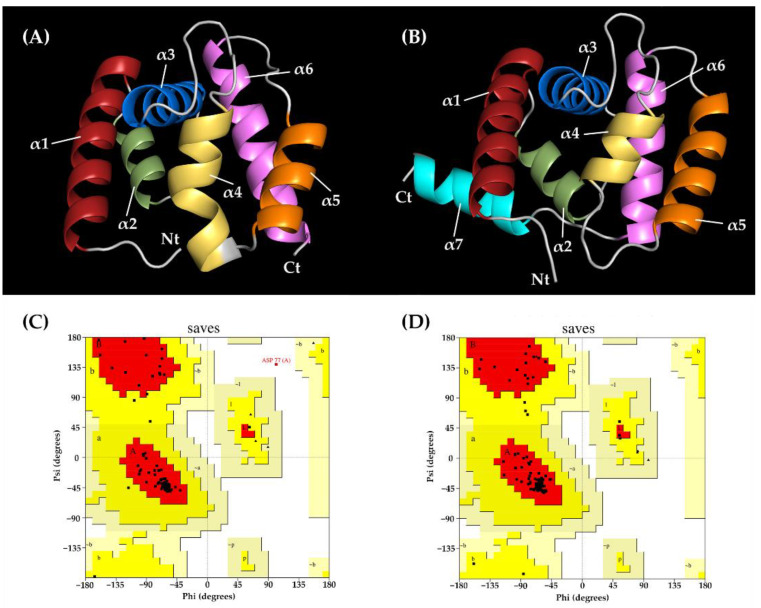
Three-dimensional structure model and Ramachandran plot of EparOBP2. (**A**) The 3D structures of EparOBP2 constructed using SWISS-MODEL. (**B**) The 3D structures of EparOBP2 constructed using trRosetta. (**C**) Ramachandran plot of EparOBP2 model constructed using SWISS-MODEL. (**D**) Ramachandran plot of EparOBP2 model constructed using trRosetta.

A 3D structural model was constructed using two different programs: SWISS-MODEL and trRosetta. The crystal structures of *Phormia regina*’s odorant-binding protein (31.25% identity) were selected as a template model for homology modeling. Only six α-helices were exhibited in this model (Figure 4A). The Ramachandran plot showed that 93.3% of the residues could be found in the most favorable region and that 5.8% of all residues were in additionally allowed regions (Figure 4C). The verified 3D results showed that 88.5% of the EparOBP2 residues scored above 0.2 (Figure S1A). The TM score of the 3D model constructed using trRosetta was 0.878, indicating that this model had correctly predicted the topology. Seven α-helices were exhibited in this model (Figure 4B). The Ramachandran plot showed that 94.0% of residues were found in the most favorable region and that 6% of all residues were in additional favorable regions (Figure 4D). The Verify 3D results also showed that 84.92% of the EparOBP2 residues scored above 0.2 (Figure S1B).

### 2.4. Expression and Purification of Recombinant EparOBP2

**Figure 5:**
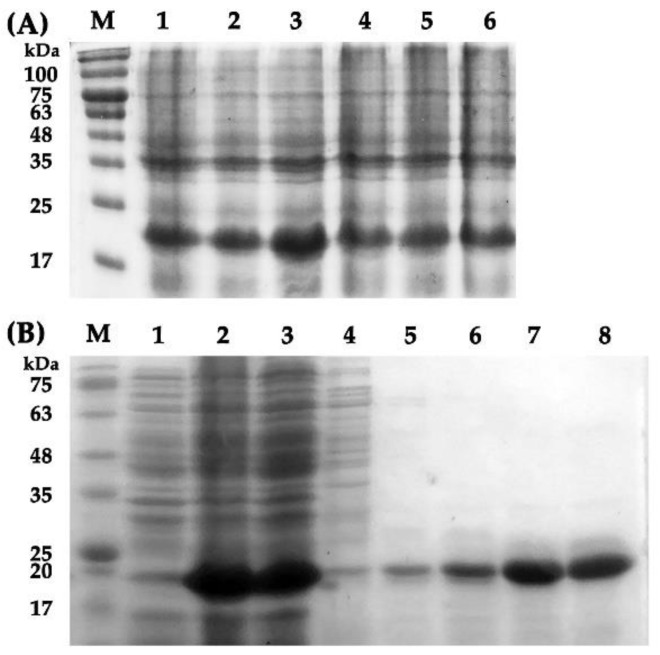
SDS-PAGE analysis of recombinant protein EparOBP2 expression and purification. (**A**): Expression levels of recombinant EparOBP2 with different induction times and IPTG concentrations. M: protein molecular weight marker; (1): the supernatant from protein samples induced by 0.1 mM IPTG over 3 h; (2): the supernatant of protein samples induced by 0.1 mM IPTG over 6 h; (3): the supernatant of protein samples induced by 0.1 mM IPTG over 9 h; (4): the supernatant of protein samples induced by 0.5 mM IPTG over 3 h; (5): the supernatant of protein samples induced by 0.5 mM IPTG over 6 h; 6: the supernatant of protein samples induced by 0.5 mM IPTG over 9 h. (**B**): SDS-PAGE analysis of the purification of recombinant EparOBP2. M: protein molecular weight marker; (1): protein sample without IPTG induction; (2): protein sample after 0.1 mM IPTG induction for 9 h; (3): protein fluid after flowing through the column; (4–8): protein samples were obtained by means of imidazole elution at different concentrations (20, 60, 100, 200, and 500 mmol/L imidazole).

Recombinant EparOBP2 was successfully isolated from the supernatant of *E. coli* BL21 (DE3) after ultrasonication. The SDS-PAGE showed that the MWs of recombinant EparOBP2 were approximately 22 kDa, which is consistent with the predicted MWs of ExPASy (Figure 5A). The proteins were then purified using Ni-NTA resin affinity chromatography (Figure 5B), and their binding properties were tested after the digestion of the His-Tag using enterokinase.

### 2.5. Fluorescence Competitive Binding Assays

**Figure 6:**
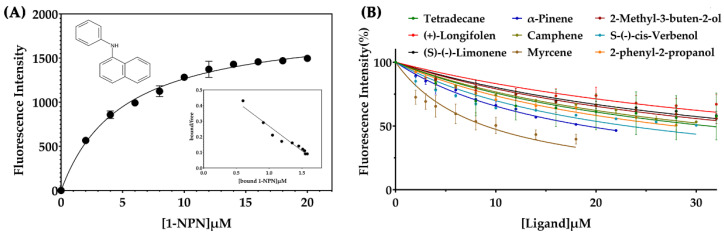
Competitive fluorescence ligand-binding assay of EparOBP2 to ligands. (**A**): Binding curve and relative Scatchard plot of EparOBP2 and 1-NPN. (**B**): Competitive binding curves of EparOBP2 with nine ligands.

To measure the binding affinity of the constants toward EparOBP2, the fluorescent reporter 1-phenyl-1-naphthylamine (1-NPN) was used as a fluorescent probe. We calculated the dissociation constant by observing and analyzing the saturation as well as the linear Scatchard plot curves. EparOBP2 bonded to 1-NPN, with a K_1-NPN_ of 4.98 μM (Figure 6). The Ki values of the nine volatiles with EparOBP2 were then calculated using the K_1-NPN_ value. Competitive fluorescence binding curves showed that the relative fluorescence intensity of the EparOBP2–1-NPN complex decreased with the addition of the nine different ligands (Figure 6). This indicated that EparOBP2 has broad binding properties toward those nine semiochemicals. The compounds myrcene and α-pinene had high binding affinities to EparOBP2, with Ki values of 7.49 and 15.87 μM, respectively (Table 1).

### 2.6. Molecular Docking

**Figure 7:**
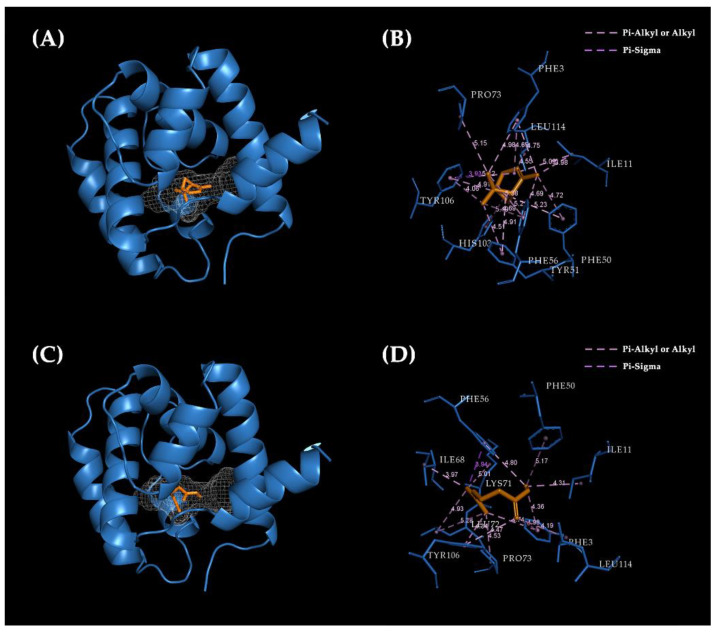
Molecular docking of EparOBP2 with α-pinene and myrcene. (**A**) 3D structure of EparOBP2 with α-pinene. (**B**) 3D predicted interaction view of EparOBP2 with α-pinene. (**C**) 3D structure of EparOBP2 with myrcene. (**D**) 3D predicted interaction view of EparOBP2 with myrcene. The binding pocket was marked by meshes.

To further validate the results of the ligand-binding assay, and to investigate the binding mode of EparOBP2 with semiochemicals, the ligands with the highest binding ability (i.e., Ki less than 20 µM) were chosen to bind with the model EparOBP2 constructed using trRosetta. These two compounds—myrcene and α-pinene—exhibited good interactions with EparOBP2, with binding energies of −6.5 and −6.4 kcal/mol, respectively. Myrcene and α-pinene tended to dock in the same binding pocket, which was located in the center of a hydrophobic tunnel (Figure 7A,C). In general, hydrophobic interactions—such as pi–alkyl, pi–sigma, and alkyl interactions—represented the main interactions between EparOBP2 and its ligands.

α-Pinene bound with Ile11 (3.98 Å), Pro73 (5.15 Å), and Leu114 (4.55 Å) through alkyl interactions. Phe3 (4.65 Å), Phe50 (4.72 Å), Tyr51 (4.51 Å), Phe56 (5.21 Å), His103 (5.49 Å), and Tyr106 (4.82 Å) were involved in pi–alkyl interactions. Moreover, Tyr106 (3.93 Å) formed a pi–sigma interaction with α-pinene (Figure 7B). During the binding of EparOBP2 and myrcene, Phe3 (5.75 Å), Phe50 (6.49 Å), Phe56 (6.26 Å), and Tyr106 (5.71 Å) were involved in pi–alkyl interactions, while Ile11 (4.78 Å), Ile68 (3.77 Å), Lys71 (5.59 Å), Leu72 (4.64 Å), Pro73 (4.92 Å), and Leu114 (4.78 Å) were bound through alkyl interactions. Myrcene showed pi–sigma interactions with Phe56 (4.91 Å) (Figure 7D). Additionally, van der Waals interactions with Tyr51 and His103 were also observed.

## 3. Discussion

Most platypodine species only infest freshly dead or dying trees [27]. *E. parallelus* is one of the few species that can successfully colonize live trees [6]. In the Hainan rubber-planting area, this species has been reported to be one of the dominant species of rubber bark beetles and represents a threat to rubber production in Hainan. Semiochemicals, including pheromones and volatile plant compounds, can influence the communication between insects both directly and indirectly. These semiochemicals have been used as effective biological agents to reduce the use of pesticides [19,28,29,30]. To clarify the molecular mechanism of EparOBP2 involved in semiochemical detection, the cDNA sequence of EparOBP2 was analyzed. The predicted mature protein EparOBP2 is a small, soluble secretory protein with a molecular mass of 16.87 kDa and a signal peptide that is 21 amino acid residues in length at the N-terminus. The EparOBP2 sequences shared a conserved OBP sequence motif found in Coleoptera (C1-X_23–44_-C2-X_3_-C3-X_36–43_-C4-X_8–12_-C5-X_8_-C6), with the only difference being the number of amino acids between the fourth and fifth cysteines. The distance between the fourth and fifth cysteines is quite varied in most insects [31]. Six conserved cysteines formed three pairs of disulfide bridges, proving that EparOBP2 could belong to the classic OBP subfamilies [32]. The 3D structural model of EparOBP2 was constructed using two modeling methods: SWISS-MODEL and trRosetta. These two modeling servers represent two distinct modeling approaches: template-based modeling, and template-free modeling, respectively [33,34,35]. As one of the most important template-based modeling methods, SWISS-MODEL builds reliable 3D models of a target protein from its amino acid sequence on the basis of an alignment with a similar protein with a known structure. The major limitation of this technique is the availability of homologous templates [36]. Only regions of the protein corresponding to an identified template can be modeled accurately. The template-free modeling relies on large-scale conformational sampling and the application of physics-based energy functions [35]. The trRosetta server is powered by deep learning and combines both de novo and template-based modeling [34]. It works well for a broad range of targets. The modeling result based on SWISS-MODEL had only six α-helices, and the last α-helix was deficient due to the limitation of the selected template’s structure. Only regions of EparOBP2 that matched the PregOBP crystal structure (PDB: 5dic.1.A) could be effectively represented. The 3D structure of EparOBP2 constructed utilizing the trRosetta method possessed higher integrity. Additionally, we assessed the quality of the two models by using SAVES v6.0. The Ramachandran plots showed a larger proportion of residues belonging to the most favored regions and additionally allowed regions. This indicated the good quality of the EparOBP2 model constructed using the trRosetta server.

The results of the fluorescence binding assay demonstrated that EparOBP2 is specifically bound to myrcene and α-pinene and has high binding affinities. Myrcene and α-pinene are both important components of volatile plant compounds [10]. A large amount of research has revealed that these two components can effectively manipulate the behavior of bark beetles [37]. For example, α-pinene plays the role of an attractant of *Dendroctonus valens*, *D. rufipennis*, and *Ips typographus* [38,39,40]. For *D. ponderosae*, host monoterpenes—including α-pinene, myrcene, and terpinolene—synergistically enhance attraction effects [41]. The exo-brevicomin released by adult females of *D. brevicomis*, combined with the myrcene released by host plants, shows attraction effects for conspecifics during the early stages of tree colonization [37]. Furthermore, myrcene and α-pinene have also been proposed to be the precursors of pheromone components based on their structural similarity with ipsdienol and verbenol [42,43,44]. Female *D. ponderosae* initiate host colonization, and males and additional females respond to two α-pinene derivatives—trans- and cis-verbenol—that are released by the pioneering females [45].

The binding properties of OBP with myrcene and α-pinene have been reported in several insects. For example, the OBP1, OBP7, OBP4, and OBP from *Anopheles gambiae* have shown strong binding affinities with α-pinene and myrcene and primarily interact with them through hydrophobic interactions [46]. AplaOBP1, which shows high binding affinities with host-tree terpenes (including myrcene), was predicted to participate in the detection of host volatiles in *Agrilus planipennis* [47]. The docking results confirmed the interactions of α-pinene and myrcene with EparOBP2. They primarily tend to bond with EparOBP2 through hydrophobic interactions, including alkyl, pi–alkyl, and pi–sigma interactions in a conserved binding pocket. Hydrophobic residues, such as Phe3, Tyr5, Ile11, Phe50, Phe56, Pro73, Tyr106, and Leu114, showed high levels of overlap and conservation, facilitating interaction with odors rather than hydrophilic residues. These amino acid residues are important in the formation of interactions between proteins and ligands. A site-directed mutagenesis technique was needed to identify the specific roles of these residues.

## 4. Materials and Methods

### 4.1. Insect Collection

Adults of *E. parallelus* were trapped using aggregation pheromones collected from the rubber forest on the Danzhou campus of Hainan University (19.51° N, 109.49° E). The whole bodies were immediately frozen in liquid nitrogen and stored at −80 °C until RNA isolation.

### 4.2. Sequencing Analysis

The EparOBP2 cDNA sequence was cloned and submitted to the NCBI GenBank database (Accession No: OQ160971). The deduced amino acid sequence was analyzed using DNAMAN (Lynnon Biosoft, San Ramon, CA, USA). SignalP V4.1 was used to predict the N-terminal signal peptide sequences (https://services.healthtech.dtu.dk/service.php?SignalP-4.1/ accessed on 20 October 2021) The online program tools ProtParam (https://web.expasy.org/protparam/ accessed on 20 October 2021), SOPMA (https://npsa-prabi.ibcp.fr/cgi-bin/ accessed on 22 December 2021), ProtScale (https://web.expasy.org/protscale / accessed on 20 October 2021), and ESPript3.0 software were used to predict the chemical and physical properties, secondary structure, and hydrophobicity scales of EparOBP2 [48]. Sequences of similar OBPs from other Coleoptera species were obtained by searching GenBank using the NCBI-BLASTp network server. Eighteen OBP sequences taken for sequence alignment and phylogenetic analysis are listed in Table S1. Multiple alignments were performed using ClustalW. The MEGA 5.2 program was employed to construct the phylogenetic trees of EparOBP2 with other OBPs by using the neighbor-joining method and a model that included the number of differences and the pairwise deletion of gaps.

### 4.3. Expression and Purification of Recombinant EparOBP2

The cDNA sequence of the EparOBP2-removed signal peptides was amplified using the designed primers. Enzymatic digestion sites were designated EcoRI and NotI. The forward primer was 5-CGGAATTCCAGGACTTACTGAAGAAC-3 (the EcoRI restriction site is underlined), and the reverse primer was 5-ATATGCGGCCGCTGATTTTGTTTCTTTTGCTGC-3 (the NotI restriction site is underlined). The purified PCR product and the pET-32a (+) vector (Solarbio, Beijing, China) were ligated and transformed into *E. coli* BL21 (DE3) cells (AxyGen, Shanghai, China). *E. coli* cells were cultured until the OD600 value reached 0.6, while β-D-1-thiogalactopyranoside (IPTG) was added to the LB medium at a final concentration of 0.1 mM. Bacterial cultures were continuously shaken at 200 rpm at 30 °C for 9 h to induce the recombinant EparOBP protein. The suspension was crushed by sonication and then separated into supernatant and sediment by centrifugation (6000 rpm, 10 min, 4 °C). Then, pET-32a (+)-EparOBP2 was purified and desalted using Ni Sepharose 6FF (Solarbio, Beijing, China) and Amicon^®^ Ultra-4 3K centrifugal filters (Merck Millipore, Darmstadt, Germany), respectively. Finally, the His-tag was removed by enterokinase, and the size and purity of the recombinant protein were analyzed using 15% SDS-PAGE.

### 4.4. Fluorescence Competitive Binding Assays

Nine semiochemicals related to the life activities of *E. parallelus* were used as candidate ligands. The binding capacities of EpraOBP2 were evaluated using an F-7000 fluorescence spectrometer (Hitachi, Tokyo, Japan). The fluorescent reporter N-phenyl-1-naphthylamine (1-NPN) was dissolved in chromatographic methanol at a concentration of 1 mM. The purified EparOBP2 was dissolved in 50 mM Tris-HCl (pH 7.4) at a final concentration of 2 μM. The fluorescence spectra were scanned between 360 and 650 nm. Data demonstrating the formation of 1-NPN–EparOBP2 complexes were obtained by the titration of 2 μM protein with aliquots of 1-NPN to final concentrations ranging from 2 to 20 μM. Prism 8 (GraphPad Software, San Diego, CA, USA) was used to calculate the dissociation constant of 1-NPN (K_1-NPN_).

To calculate the binding ability of EparOBP2 with semiochemicals, different odor compounds were used to replace the 1-NPN from the EparOBP2/1-NPN complex. The odor ligands were dissolved in chromatographic methanol at a concentration of 1 mM and added as aliquots to the protein solution. The spectral results were obtained, and the dissociation constants of the competitors were calculated with the formula Ki = IC_50_/(1 + [1-NPN]/K_1-NPN_), where 1-NPN represents the free concentration of 1-NPN, while IC_50_ represents the ligand concentration displacing 50% of the fluorescent reporter.

### 4.5. Structure Modeling and Molecular Docking

To predict the three-dimensional (3D) structure of EparOBP2, a modeling approach based on homology modeling and deep learning was used. The online protein structure homology modeling server—the SWISS-MODEL prediction algorithm (https://swissmodel.expasy.org/ accessed on 10 June 2022)—was used with the selected template’s PregOBP crystal structure (PDB: 5dic.1.A). Another web-based platform—the transform-restrained Rosetta (trRosetta) server (https://yanglab.nankai.edu.cn/trRosetta/ accessed on 14 June 2022)—was also used to predict the 3D model of EparOBP2 based on multiple sequence alignment (MSA) and direct energy minimization. The 3D conformer structures of the semiochemicals were downloaded from the chemical compound databases ZINC (https://zinc.docking.org/ accessed on 26 May 2022) and PubChem (https://pubchem.ncbi.nlm.nih.gov/ accessed on 26 May 2022). Quality assessment of the 3D model was performed using SAVES v6.0 (https://saves.mbi.ucla.edu/ accessed on 16 June 2022). POCASA 1.1 (https://g6altair.sci.hokudai.ac.jp/g6/service/pocasa/ accessed on 22 June 2022) and DeepSite (https://playmolecule.com/deepsite/ accessed on 22 June 2022) were used to perform pocket predictions. AutoDock Vina 1.1.2 and AutoDock Tools 1.5.6 (the Center for Computational Structural Biology, La Jolla, CA, USA) were used to find the potential binding mode between EparOBP2 and the ligands. The default parameters were used for molecular docking. The binding affinity score was calculated on the basis of the potential energy changes around the binding pocket during protein–ligand interactions. A lower score corresponded to a stronger binding ability. Visual structure analysis was performed using PyMOL Viewer (http://www.pymol.org/ accessed on 5 June 2022) and Discovery Studio Visualizer (BIOVIA, San Diego, CA, USA).

## 5. Conclusions

In this study, the cDNA sequence of EparOBP2 was cloned and analyzed. The matured EparOBP2 protein consisted of seven α-helices, creating a hydrophilic binding pocket. EparOBP2 demonstrated high binding ability with α-pinene and myrcene. The docking results confirmed that the interactions and contributions of this key amino acid were primarily hydrophobic residues. This study provides evidence that EparOBP2 may be involved in the chemoreception of semiochemicals, and that it can successfully contribute to the integrated management of *E. parallelus.*

## Figures and Tables

**Table 1:** Binding affinities of different ligands to EparOBP2.

Ligands	Formula	2D Structure	CAS Number	IC50 (μM)	Ki (μM)
Myrcene	C_10_H_16_	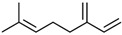	123-35-3	8.991	7.49
S-(-)-cis-Verbenol	C_10_H_16_O	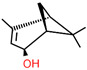	18881-04-4	23.18	19.30
2-Methyl-3-buten-2-ol	C_5_H_10_O	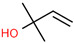	115-18-4	37.9	31.56
α-Pinene	C_10_H_16_	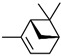	80-56-8	19.06	15.87
(S)-(-)-Limonene	C_10_H_16_	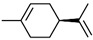	5989-54-8	40.44	33.68
(+)-Longifolene	C_15_H_24_	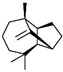	475-20-7	49.61	41.31
Tetradecane	C_14_H_30_	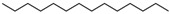	629-59-4	31.23	26.00
2-Phenyl-2-propanol	C_9_H_12_O	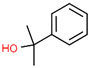	617-94-7	27.93	23.26
Camphene	C_10_H_16_	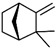	79-92-5	32.94	27.43

## Data Availability

The data that support the findings of this study are available from the corresponding author upon reasonable request.

## Supplementary Materials

The following supporting information can be downloaded at: https://www.mdpi.com/article/10.3390/ijms24021714/s1.
