# Supplementary figures and images for: The Sequence Characteristics and Binding Properties of the Odorant-Binding Protein 2 of Euplatypus parallelus to Semiochemicals
Source: Int J Mol Sci. 2023 Jan 15;24(2):1714. doi: 10.3390/ijms24021714 (PMC9863716; doi:10.3390/ijms24021714)

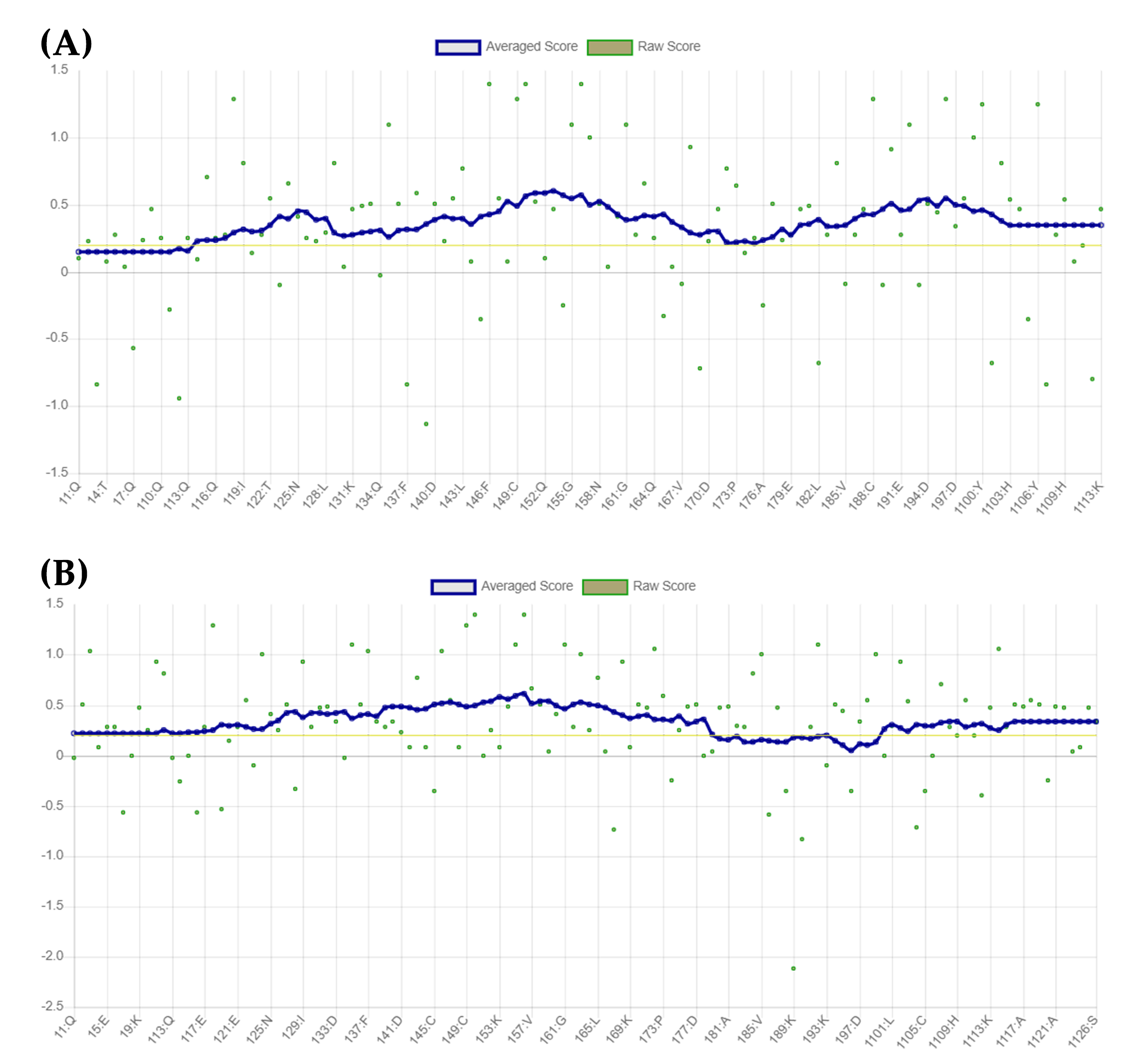

Supplement: Supplementary file 1 [file ijms-24-01714-s001.zip › Figure S1.tif]
